# Meta-analysis of estrogen response in MCF-7 distinguishes early target genes involved in signaling and cell proliferation from later target genes involved in cell cycle and DNA repair

**DOI:** 10.1186/1752-0509-5-138

**Published:** 2011-08-30

**Authors:** Vidhya Jagannathan, Marc Robinson-Rechavi

**Affiliations:** 1 Department of Ecology and Evolution, University of Lausanne, Lausanne, CH-1015, Switzerland; 2 Evolutionary Bioinformatics, Swiss Institute of Bioinformatics, Lausanne, CH-1015, Switzerland

**Keywords:** microarray, meta-analysis, estrogen, breast cancer, pathways

## Abstract

**Background:**

Many studies have been published outlining the global effects of 17β-estradiol (E2) on gene expression in human epithelial breast cancer derived MCF-7 cells. These studies show large variation in results, reporting between ~100 and ~1500 genes regulated by E2, with poor overlap.

**Results:**

We performed a meta-analysis of these expression studies, using the Rank product method to obtain a more accurate and stable list of the differentially expressed genes, and of pathways regulated by E2. We analyzed 9 time-series data sets, concentrating on response at 3-4 hrs (early) and at 24 hrs (late). We found >1000 statistically significant probe sets after correction for multiple testing at 3-4 hrs, and >2000 significant probe sets at 24 hrs. Differentially expressed genes were examined by pathway analysis. This revealed 15 early response pathways, mostly related to cell signaling and proliferation, and 20 late response pathways, mostly related to breast cancer, cell division, DNA repair and recombination.

**Conclusions:**

Our results confirm that meta-analysis identified more differentially expressed genes than the individual studies, and that these genes act together in networks. These results provide new insight into E2 regulated mechanisms, especially in the context of breast cancer.

## Background

Estrogens are fundamental to the function of the female reproductive system, and have also been shown to regulate diverse cellular processes in the central nervous system, the cardiovascular system, and bone metabolism [1]. Estrogens play a key role in proliferation and differentiation of healthy breast epithelium, but also contribute to the progression of breast cancer by promoting the growth of transformed cells [2]. The predominant effect of estrogen is mediated through its interaction with two intracellular estrogen receptors, ERα and ERβ. ERα notably is strongly expressed in 80% of breast cancers [3]. Once estrogen is bound to ERα, the receptor dimerizes and associates with chromatin. ERα homodimers bind either directly to a DNA sequence motif, the estrogen response element [4-6], or indirectly via tethering to other transcription factors such as specificity protein 1 (Sp1) [7,8], activating protein 1 (AP-1) [9,10], or nuclear factor kappa b (NF-κB) [11].

17β-Estradiol (E2) is the most prevalent intracellular estrogen. Tiled or cDNA microarrays have been abundantly used for the global analysis of 17β-Estradiol (E2) responsive genes. Several studies have profiled the transcriptional response to E2 in cultured cells, particularly MCF-7 cells, and have uncovered comprehensive features of E2 regulated gene expression. At early time points following E2 induction a greater proportion of genes are up regulated, while at later time points more genes are down regulated [12]. It has been hypothesized that early ERα-mediated down regulation might be due to squelching, while the increase in the number of down regulated genes at later time points might depend on the up regulation of the corepressor nuclear receptor interacting protein 1 (NRIP1), which mediates the repression of ERα target genes [5]. Interestingly, only about 20% to 30% of the E2-regulated genes represent direct targets [13], as defined by treatment with the protein translation inhibitor cyclohexamide.

There is considerable variation in the results generated by different laboratories, even for the same cell type and hormone treatment[14]. Estimations of the number of E2-regulated genes range from ≈100 to ≈1500[14]. As noted by Cheung et al., [15] the differences between studies can be due to the origin and growth conditions of the cell lines, the length of hormone treatment, the array platforms used, or the bioinformatics methods used for data analysis.

Meta-analysis allows combining the results of several studies, to provide a global picture, with statistical support, and hopefully better power and specificity than each of the original studies. Several meta-analysis applications for microarrays have been proposed [16-18]. Two methods have been most frequently applied: one is to combine p-values, and the other is to combine effect sizes. Combining p-values has been very useful in obtaining more accurate estimates of significance. Choi et al [16]have shown that combining effect sizes can take into account inter-study variation. Here we have used the Rank product [19] method to detect differentially expressed genes in response to E2 treatment in MCF-7 cell lines, by integrating multiple array datasets from two different platforms across laboratories. This method detects genes that are consistently highly ranked in several replicated experiments, independently of their numerical intensities. It generates a single significance measurement for each gene in the combined study.

## Results

### Data set selection and processing

Microarray Data was downloaded from GEO (http://www.ncbi.nlm.nih.gov/geo/) [20]. Nine time course studies were used for the meta-analysis. Two common time points among the different datasets were selected for further analysis: 3 to 4 hours of treatment (early) and 24 hours of treatment (late). In total we had 4 datasets that were used for the early time point, and 7 datasets that were used for the late time point (tables 1 and 2).

**Table 1:** Studies used for meta-analysis

GEO accession	Reference	Early	Late
GSE3834	[34]	*	*
GSE9936	[35]	*	*
GSE11324	[36]	*	
GSE5840	[37]	*	
GSE3529	[38]		*
GSE4006	[36]		*
GSE4025	[39]		*
GSE6800	[37]		*
GSE8597	[40]		*

**Table 2:** Conditions of treatment and expression measure of each study

GSE dataset	E2	Normalization
GSE3529	10 nM	RMAExpress
GSE3834	10 nM	RMAExpress
GSE4006	10 nM	GCRMA
GSE4025	10 nM	GCRMA
GSE5840	10 nM	Raw scaled signal count
GSE6800	1 nM	RMA
GSE8597	25 nM	RMA
GSE9936	6 nM	RMA
GSE11324	100 nM	RMA

Eight of the 9 studies were provided with data normalized by the Robust Multi-Array Average (RMA)[21] method, or the related Guanine Cytosine Robust Multi-Array Analysis (GCRMA)[22] method. For one study raw scaled signal count values were provided. Rank product method does not require the recalculation of the normalized expression values. Thus we use the data processed according to what was considered best practice in each lab.

For the meta-analysis, the probesets found in common across the Affymetrix U133 GeneChip family was used. There are 22,277 common probesets between the two U133a and U133 plus 2.0, which map to 13,186 genes.

### Meta-Analysis of gene expression data

The Rank product method was chosen, as non-parametric analyses are more robust in general, which is an important concern in comparing experiments done in different laboratories. The Rank product method has been shown to give good results on microarray data [18]. Out of 22,277 probe sets, we identified 1206 at the early time point and 4193 at the late time point, with a false discovery rate (called percentage of false positive predictions or pfp, see methods) ≤ 0.05. The high proportion of genes with low p-values indicates that many more genes are found to be differentially expressed than expected by chance. By mapping the probe sets to genes, we have identified 991 unique genes differentially expressed at the early time point and 3234 unique genes that are differentially expressed at the late time point. All results are available in Additional File 1.

**Figure 1:**
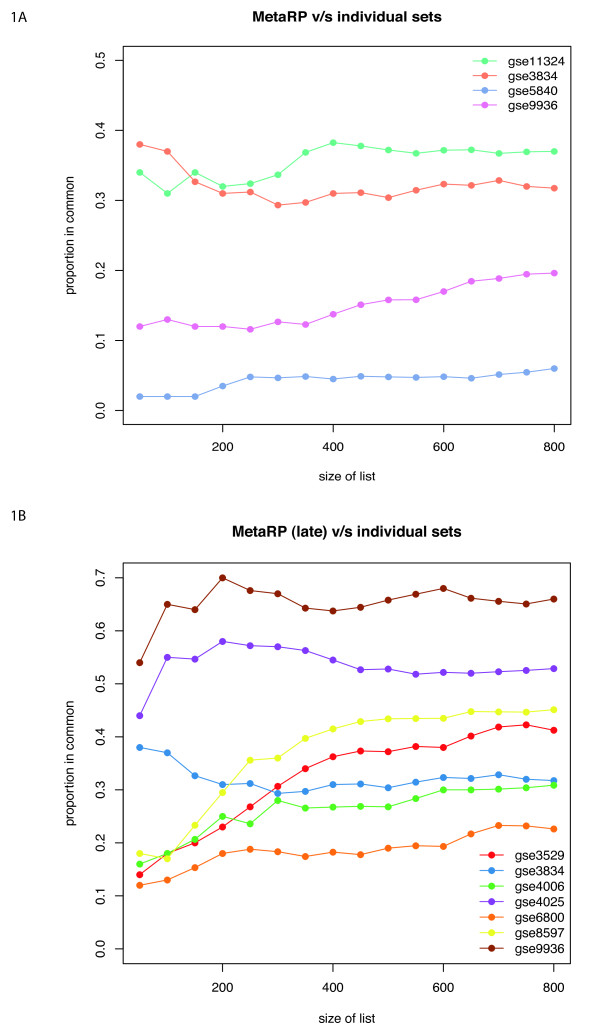
**CAT plots**. Concordance at the top plots comparing ranked probe set lists from of early time point (3-4 hrs) (A), and for late time point (24 hrs) (B).

We compared the proportion of top genes identified in individual datasets with the top genes from the meta-analysis, using Correspondence At the Top (CAT) plots [17]. CAT plots quantify the consensus of two lists, based on ranks. For example if two lists have 40 genes out of the first 100 in common, the their consensus at rank 100 will be 40%. In Figure 1 the CAT plots are shown for the top 800 probes (400 up-regulated and 400-down regulated) at each time point. These plots show that no single study determines or influences the final meta-analysis gene list. Table 3 also shows that the meta-analysis is able to identify more genes at the same pfp level, demonstrating an increased statistical power.

**Table 3:** Average number of genes identified at different pfp levels and integration driven discovery rate (IDD) in meta-analysis(shown in parenthesis)

Pfp Value	Early Single	Early Meta	Late Single	Late Meta
0.00001	101	147(0.32)	680	1271(0.46)
0.0001	112	210(0.47)	979	1673(0.41)
0.001	234	350(0.33)	1795	2223(0.2)
0.005	470	521(0.10)	2834	2803

"Single" reports the union of all genes recovered by any single study at a given pfp value.

### Top Genes

Tables 4 and 5 show the top 10 unique genes from the meta-analysis for the early time point and their ranks in the individual datasets. The tables also show the ranks of the union of all top 10 genes from individual datasets and meta-dataset, to illustrate the specificity of the meta-analysis relative to individual experiments.

**Table 4:** Comparison of top 10 early up regulated genes

Genes	GSE11324	GSE3834	GSE5840	GSE9936	Meta
RET	4	6	19	17	1
GREB1	1	1	*	26	2
CXCL12	12	4	*	33	3
MYBL1	3	5	*	85	4
CA12	15	18	48	19	5
IGFBP4	42	*	30	7	6
PMAIP1	20	27	*	14	7
RAPGEFL1	163	*	1	*	8
WWC1	171	32	8	102	9
SGK3	2	19	*	*	10
TUBB2B	*	*	3	*	13
STC2	10	*	*	15	14
EGR3	143	2	*	215	15
NRIP1	9	*	*	44	18
SGK1	5	*	*	347	19
AREG	138	9	24	61	23
MYC	19	7	*	104	24
HSPB8	*	3	56	62	25
PGR	153	*	2	*	29
FADS1	*	*	*	5	30
IL17RB	*	*	*	4	39
PLOD2	6	*	*	131	40
AMD1	7	*	*	*	41
RASGRP1	11	10	*	*	42
TNP2	*	*	7	*	49
MYB	8	*	*	181	57
HOXC4	*	*	*	9	65
CHST8	*	*	6	128	91
SERPINB9	*	*	*	3	113
GP5	*	*	10	*	123
FCGR2B	*	*	4	*	141
TGM2	*	*	5	*	153
TFF1	313	*	*	8	210
TFF2	*	*	9	*	217
HOXC6	*	*	*	1	282
PLAC1	*	*	*	2	290

The column gene includes a union of top 10 regulated genes from individual studies and meta-analysis. The ranks of these genes are indicated in the individual study and in the meta analysis studies. The asterisk * indicates that the gene was not included in the significant list (pfp ≤ 0.05).

**Table 5:** Comparison of top 10 late down regulated genes

Gene	M	gse3529	gse3834	gse4006	gse4025	gse6800	gse8597	gse9936
IL17RB	1	*	*	8	5	*	12	2
TFF1	2	*	*	92	19	*	655	1
CDK1	3	*	59	28	13	*	57	5
MYBL1	4	1	6	31	174	*	1	162
CDC20	5	43	3	*	15	*	310	3
CXCL12	7	15	8	135	68	*	17	57
PBK	8	39	16	*	18	*	23	33
RAB31	9	29	*	14	4	*	93	25
KIF2C	10	*	63	*	7	*	226	4
PDZK1	419	2	25	57	*	*	414	*
GREB1	19	3	1	1	194	1	62	323
EGR3	184	4	42	41	*	4	19	*
AREG	*	5	7	38	*	21	46	719
NPY1R	57	6	*	13	*	*	187	96
MSMB	1650	7	*	*	*	*	*	*
RASGRP1	*	8	*	*	*	*	1020	*
FABP5	*	9	*	*	*	23	98	*
CELSR2	136	10	33	89	*	26	313	402
DHRS2	294	*	2	21	*	*	639	542
PTTG1	29	*	4	*	77	*	346	14
KIF20A	18	*	5	*	17	*	304	7
PLK1	74	*	9	*	45	*	389	44
CENPA	15	71	10	*	39	*	270	9
GJA1	*	*	*	2	*	*	*	*
WISP2	549	*	67	3	*	*	342	*
ASCL1	704	*	*	4	*	*	*	400
OLFML3	65	*	*	5	114	*	*	29
EPB41L3	605	*	*	6	*	*	*	357
PEG10	567	*	*	7	*	*	*	505
PGR	628	*	*	10	*	*	94	*
PRIM1	12	*	*	*	1	*	56	15
SLC7A5	107	*	*	*	2	24	*	132
PLAC1	17	*	*	132	3	*	13	53
TPD52L1	14	32	*	97	6	19	363	39
KIF4A	21	*	*	*	8	*	148	8
TPX2	36	*	40	*	10	*	244	34
SLC26A2	293	*	66	109	*	2	290	*
PRSS23	101	21	*	61	61	3	675	246
PTP4A1	709	*	*	*	*	5	*	966
CA12	30	55	*	65	44	6	249	60
HSPB8	1739	40	32	*	*	7	*	*
TSKU	*	*	*	*	*	9	*	*
SGK3	93	22	*	23	*	*	2	778
UGT2B15	112	*	*	26	*	*	3	227
TMPRSS3	220	16	*	56	*	22	4	*
PCP4	255	*	*	*	*	*	5	867
MCM10	92	41	*	*	200	*	6	243
DSCC1	132	62	*	*	*	*	7	453
EXO1	338	*	*	*	*	*	8	*
DTL	53	*	*	79	261	*	9	95
CENPI	463	*	*	*	*	*	10	*
DLGAP5	25	*	17	*	54	*	159	10

The column gene includes a union of top 10 regulated genes from individual studies and meta-analysis. The ranks of these genes are indicated in the individual study and in the meta analysis studies. The asterisk * indicates that the gene was not included in the significant list (pfp ≤ 0.05).

The top early genes from the meta-analysis include many well known direct targets of ERα and ERβ [3]. The most significant ones include TFF1, CCND1, IGFBP4, C3, ADORA1, GREB1, and MYC, which have been shown by candidate gene analysis to be estrogen regulated genes in breast cancer cell lines.

**Figure 2:**
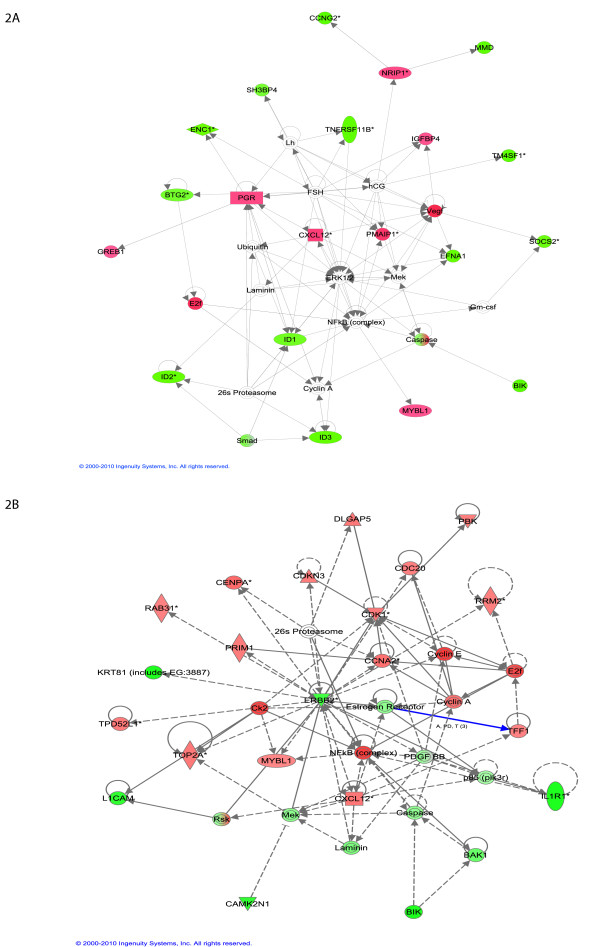
**Highly connected network of top 30 ranked genes at early time point (A)**. and late time point (B) as identified with IPA. Green node color indicates down regulation and red node color indicates up regulation. White color indicates nodes that are not included in the dataset but were assimilated into network by interaction with other molecules. Direct and indirect interactions between genes are denoted with solid and dashed lines respectively. The style of the *arrows *indicates specific molecular relationships and the directionality of the interaction (A acts on B). The *shapes *are indicative or the molecular class (*i.e*. protein family). Horizontal oval = transcription regulator, vertical diamond = enzyme, and circle = other.

Moreover, the top genes from both the early and late time points form highly connected networks (Figure 2). These interactions show the functional relevance of our results, and point to the importance of networks in interpreting E2 signaling in MCF-7. We can already see in these data important differences between the early and late regulated genes, with the top early genes involved in cellular growth and proliferation. The early network notably shows up-regulation of the nuclear repressor protein NRIP1/RIP140[5], which is then involved in the down-regulation of cyclin G2 (CCNG2) and of monocyte-to-macrophage differentiation-associated protein (MMD) (Figure 2A). The late gene network is centered on ERBB2, a member of the epidermal growth factor (EGF) family of receptor tyrosine kinases, and one of the major molecular prognostic and predictive markers in breast cancer (Figure 2B). ERBB2 is known for its role in cell proliferation, growth, and apoptosis. This network also includes NF-kappaB, which plays a key role in the aggressiveness of breast cancer cells [23,24].

### Pathway analysis: Early time point

**Figure 3:**
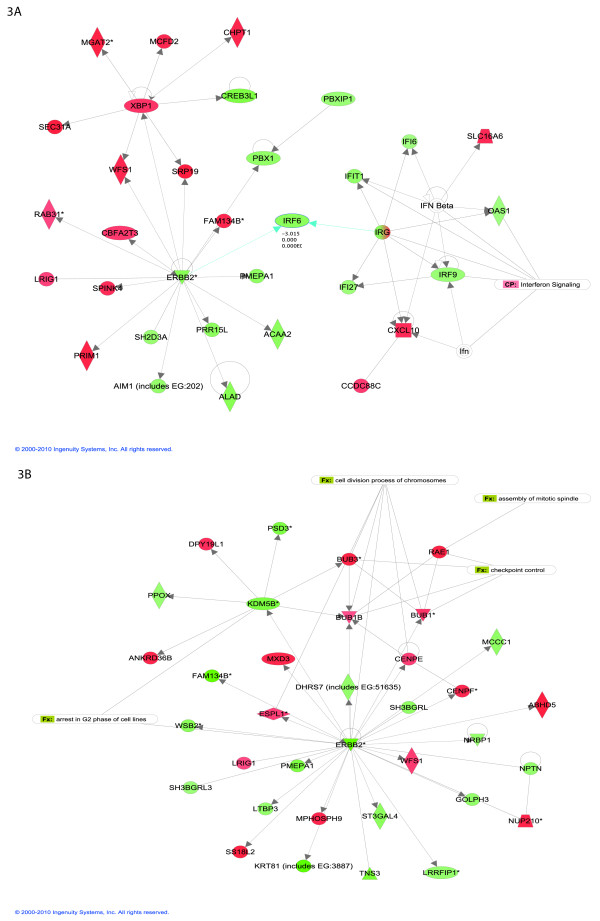
**Top networks with IPA analysis at early time point (A)**. and late time point (B). Green node color indicates down regulation, and red node color indicates up regulation. White color indicates nodes that are not included in the dataset but were assimilated into the network by interaction with other molecules. Direct and indirect interactions between genes are denoted with solid and dashed lines respectively. The style of the *arrows *indicates specific molecular relationships and the directionality of the interaction (A acts on B). The *shapes *are indicative or the molecular class (*i.e*. protein family). Horizontal oval = transcription regulator, vertical diamond = enzyme and circle = other.

We have found 20 biological networks with a significant involvement of the genes differentially expressed at the early time point. Figure 3 shows the top network, which is consistent with the network found using the top 30 genes only: again, ERRB2 plays a central role. This network is annotated "infection mechanism, cellular growth and proliferation, genetic disorder". The interferon signaling pathway, which is involved in negative regulation of cellular proliferation and induction of cellular apoptosis, is down regulated. Of note, IRF6 is suggested to regulate epithelial cell differentiation, and several IRF family members are known to harbor tumor suppressive functions. We find IRF members IR6 and IRF9 to be down regulated in MCF-7, which adds more evidence to the hypothesis that down-regulation of the interferon signaling pathway might be important for uncontrolled cellular proliferation[25].

The other top scoring networks at the early time point contain genes involved in cell growth, proliferation, DNA replication, protein amino acid metabolism, RNA and protein trafficking, cellular movement, and lipid metabolism. The merged image of the top three networks indicates three nodes centered on ERBB, cMYC and FOS, all key regulators of proliferative responses (see Additional File 2). In addition, several proteins involved in protein translation and metabolism are up regulated, including seven mitochondrial ribosomal proteins.

**Figure 4:**
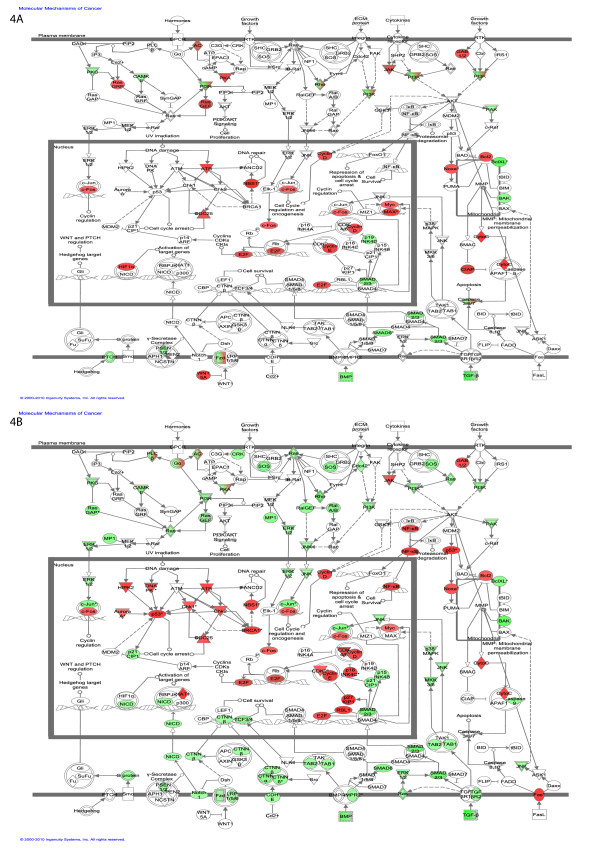
**Top Canonical pathways regulated**. (A) Early time point (B) Late time point. Green color denotes the percentage of genes in the pathway that are down regulated and red color denotes the percentage of genes that are up regulated.

In addition to searching for relations amongst the differentially regulated genes, we can map them to very well established ("canonical") pathways. In this view, the early genes appear involved in signaling pathways such as wnt/b-catenin signaling, RAR activation and VDR activation (Figure 4A).

### Pathway analysis: Late time point

**Figure 5:**
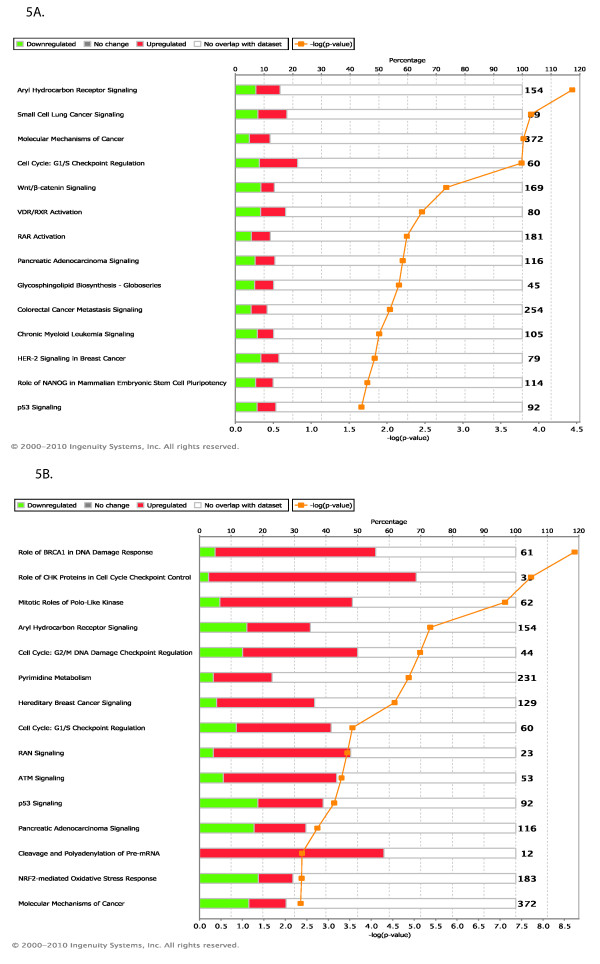
**Molecular mechanism of Cancer**. (A) The genes regulated at the early time point. (B) The genes regulated at the late time point. Green node color indicates down regulation, and red node color indicates up regulation. White color indicates nodes that are not included in the dataset but were assimilated into network by interaction with other molecules. Direct and indirect interactions between genes are denoted with solid and dashed lines respectively. The style of the *arrows *indicates specific molecular relationships and the directionality of the interaction (A acts on B). The *shapes *are indicative or the molecular class (*i.e*. protein family). Horizontal oval = transcription regulator, vertical diamond = enzyme and circle = other.

At the late time point the top network is annotated "cellular assembly and organization, cell cycle, DNA replication, recombination and repair". Figure 4 shows that the ERBB2 network is now involved in interaction with genes involved in cell division and cell cycle. The 5 top scoring gene networks share several common genes regulated at both time points, although they sometimes change in opposite directions. This similarity between time points indicates that most of the major gene expression changes start to take place as early as 3 hrs. This is clear comparing the early and late response of the genes that are involved in molecular mechanisms of cancer (Figure 5).

Genes that are down regulated at the late time point also include a large proportion of structural and cell adhesion genes, such as collagens, keratins, and other intermediate filament proteins. Other down regulated genes that are potentially involved in the development of cancer include superoxide dismutase 3 (SOD3), cyclin D2 (ccnd2), transthyretin (Ttr), bone morphogenetic protein 2 (BMP2) and matrix metalloproteinase 2 (Mmp2).

Finally, the late genes can be mapped to canonical pathways involved in breast cancer, and cell cycle and division (Figure 4B).

### Comparison with the GEMS dataset

**Figure 6:**
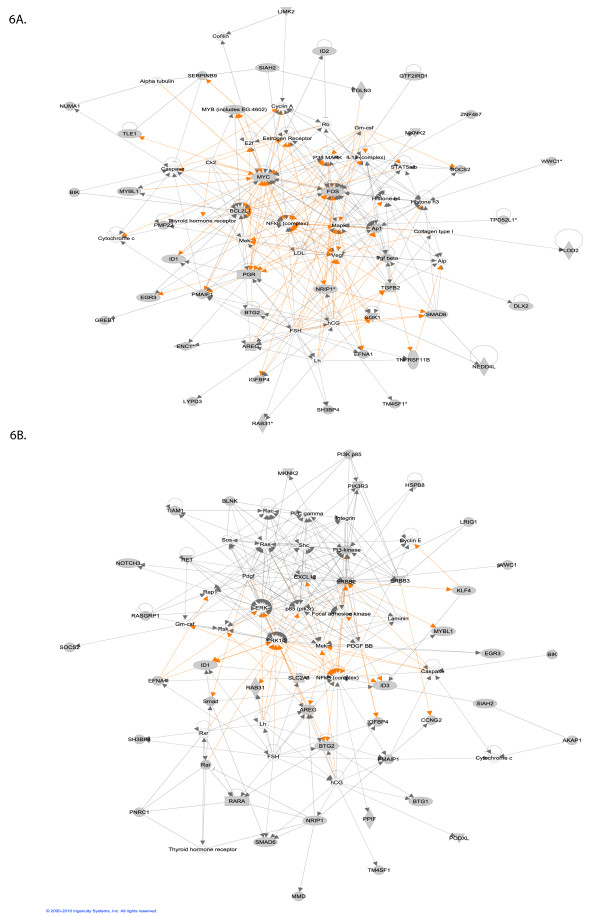
**Merged network created from the top 100 (50 down regulated and 50 upregulated) differentially expressed genes using (A) Rank product algorithm, (B) the GEMS list**.

A previous meta-analysis of E2 treated MCF cell lines was done by Ochsner et al. [26]. They used a parametric (weighted t-stats) method to obtain a gene expression meta-signature (GEMS) for E2-regulated gene expression in MCF-7 cells. We compared the pathway analysis for the top 50 down regulated and top 50 up regulated genes of the early time point of each study (Figure 6). Table 6 shows the list of central genes in each resulting network. Curiously, the GEMS derived network for early genes resembles more the networks derived from late genes by the Rank product method. Notably, it is centered on ERBB2 and ERK. Another difference is that the Rank product produces more highly connected genes which have been shown in the literature to be regulated by ERα, than GEMS. This includes ERα itself, which is not a top gene in the GEMS analysis. Also notable is the vascular endothelial growth factor (VEGF), which is central in the network derived from Rank product, but is not detected significantly in any individual study, nor by the GEMS analysis. VEGF expression is strongly induced as an immediate early response to E2 induction in vivo (uterine epithelial cells), mediated by ERα and hypoxia-inducible factor (HIF1α) [27].

**Table 6:** Genes with most connections in networks from figure 6 and their number of connections

Early probesets from Rankproduct		Early Probeset from GEMS	
**Gene**	**# of Connections**	**Gene**	**# of Connections**
MYC	31	ERBB2	25
FOS	29	ERK	21
BCL2L1	18	CXCL2	16
Mapk	15	NFkB complex	19
NFkB complex	16	Focal adhesion kinase	11
PGR	11		
Estrogen Receptor	8		
AP1	8		
NR1P1	7		

Thus it seems that, while the GEMS analysis does detect relevant E2 regulated genes, it provides a less complete picture than the Rank product method does.

## Discussion

We have identified 1206 probe sets and more than 10 pathways whose expression is significantly different between control and E2 treated MCF-7 cells 3-4 hrs after treatment, and 4193 probe sets and more than 20 pathways 24 hrs after treatment.

Although very little overlap exists on the gene level between the nine expression profiling studies, the meta analysis finds common functional processes and pathways. Table 3 shows that most of the genes identified in individual studies were also identified in the meta-analysis. The rank product algorithm returns a robust ranking for all the top genes, leading to higher reproducibility and increased specificity.

It should be noted that all the datasets we used were based on the Affymetrix platform. A recently available dataset [28], using Illumina Beadchip, provides very similar results to our analysis. Another very recent dataset [29], using GRO-seq, although produced with a different focus on transient early response to E2, also provides consistent results. Thus it does not seem that our results are platform-dependent.

We have used pathway analysis tools to understand the relationship between the regulated genes. Known targets of ERα, such as MYC, ERBB2, and ESRα, are central genes at early and late time points. This shows that the statistically significant genes are biologically relevant, and that our analysis captures key aspects of the underlying physiology.

Aryl hydrocarbon receptor (AhR) is a ligand activated transcription factor involved in xenobiotic metabolism and in mediating the toxic effects of dioxin-like compounds. Crosstalk has been observed between AhR and ER, specifically with respect to ER signaling [30]. ERα has been reported to have a positive role in AhR signaling. We find this pathway highly regulated at both time points.

The top canonical pathways regulated early are involved in cellular growth, development and proliferation, whereas those regulated late are involved in DNA replication, recombination and repair, cell cycle and cell death. These canonical pathways were not evident from any of the individual studies. Another difference between early and late response to E2 is early regulation of transcription regulators, vs. late regulation of kinases and transporters.

At the late time point we also see several genes common with the SV40T/t-antigen cancer signature identified by Deeb et al.[31] for human breast, prostate and lung carcinomas. These include genes encoding 10 centromere proteins, 8 cyclins, 15 cell division cyle proteins, 7 kinesin-like family proteins, 7 multiple minichromosome maintenance-deficient proteins, and other proliferation-related proteins and signal transduction proteins.

## Conclusions

It is important to aggregate as much high-quality data as possible to minimize sources of bias influencing gene expression studies. The rank product methodology favors genes that are consistently top ranked among replicates. Therefore in the future other high quality datasets can be integrated into this work, with minimum effort. An interesting perspective will be to explore the predictive power for clinical outcome [32] of breast cancer, of the gene set from meta-analysis, to the gene sets from individual studies.

## Methods

### Meta-Analysis

The human genome U133A platform contain >22,000 probe sets and the human genome platform U133 Plus 2.0 array contains an additional >31,000 new probe sets, giving a total of >54,000 probe sets. For the meta-analysis, the probesets found in common across the Affymetrix U133 GeneChip family was used. There are 22,277 common probesets between the two U133a and U133 plus 2.0, that map to 13,186 genes.

The statistical significance of the results was evaluated by the non-parametric algorithm 'Rank products' [33], available as the 'RankProd' package at Bioconductor (http://www.bioconductor.org) [19]. This method detects genes that are consistently highly ranked in several replicated experiments, independently of their numerical intensities. This method ranks each feature within an experiment based on that features' score (e.g., Log expression values), and then combines these ranks, instead of combining the data or p-values. The results are provided in the form of P values defined as the probability that a given gene is ranked in the observed position by chance. A list of differentially regulated probe sets were selected based on the estimated percentage of false positive predictions (pfp)[33], which is equivalent to a false discovery rate. The pfp is calculated using a permutation based procedure (100,000 permutations were conducted). Genes with a pfp of less than 0.05 were selected for further investigation. As the rank-based procedure is non-parametric, it does not make assumptions about the model and the parameters from which the data came.

### Evaluation of meta-analysis

We evaluated the performance of the Rank product algorithm using Correspondence At the Top (CAT) plots [17], which determine the proportion of genes in common between experiments as a function of list size. To generate CAT plots we use the lists of top genes for each of the study including the meta studies and plot proportion in common against the list size. We also used integration-drive discovery (IDD) to measure the number of extra genes identified by meta-analysis, compared with the union set of all individual studies at the same pfp threshold value.

### Functional analysis

Functional classification analysis was done using the Ingenuity Pathway Analysis software (version 8.5). The "Core Analysis" was used, with a focus on canonical signaling pathways. Core Analysis allows for a rapid assessment and interpretation of large and small datasets in the context of biological processes, pathways, and molecular networks. In short, for a given function or pathway, the statistical significance of pathway enrichment is calculated using a right tailed Fisher's exact test based on the number of genes annotated, the number of genes represented in the input dataset, and the total number of genes being assessed in the experiment. A pathway was deemed significant if the p-value of enrichment was ≤ 0.01. The p-value is adjusted for multiple comparisons using FDR correction.

## Abbreviations

ERα: Estrogen Receptor alpha; pfp: Percentage of False positive Predictions; IPA: Ingenuity Pathway Analysis; CAT: Correspondence At the Top.

## Authors' contributions

VJ performed data analysis and writing the manuscript. MRR contributed to research design, writing and discussion of the results. Both authors read and approved the final manuscript.

## Supplementary Material

Additional file 1 **Results of the meta-analysis**. Ranking of genes following the meta-analysis, with meta-analysis scores and p-values. Click here for file

Additional file 2 **Supplementary Figure: merged networks for early genes**. Merged top three networks with IPA analysis at early time point. Legend as Figure 3. Click here for file
