# Supplementary figures and images for: Meta-analysis of estrogen response in MCF-7 distinguishes early target genes involved in signaling and cell proliferation from later target genes involved in cell cycle and DNA repair
Source: BMC Syst Biol. 2011 Aug 30;5:138. doi: 10.1186/1752-0509-5-138 (PMC3225231; doi:10.1186/1752-0509-5-138)

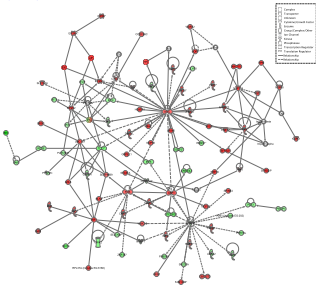

Supplement: Additional file 2 — Supplementary Figure: merged networks for early genes. Merged top three networks with IPA analysis at early time point. Legend as Figure 3. [file 1752-0509-5-138-S2.PDF]
